# Dopamine D_2_ Antagonist-Induced Striatal *Nur77* Expression Requires Activation of mGlu5 Receptors by Cortical Afferents

**DOI:** 10.3389/fphar.2012.00153

**Published:** 2012-08-14

**Authors:** Jérôme Maheux, Michel St-Hilaire, David Voyer, Emanuele Tirotta, Emiliana Borrelli, Claude Rouillard, Pierre-Paul Rompré, Daniel Lévesque

**Affiliations:** ^1^Faculté de Pharmacie, Université de MontréalMontréal, QC, Canada; ^2^Department of Psychiatry and Neuroscience, Laval University and Neuroscience Unit, Laval University Hospital Research CentreQuebec, QC, Canada; ^3^Department of Microbiology and Molecular Genetics, University of California IrvineIrvine, CA, USA; ^4^Department of Psychiatry, Faculty of Medicine, Université de MontréalMontreal, QC, Canada

**Keywords:** antipsychotic drugs, neuroleptics, *Nr4a1*, transcription factor, organotypic culture, glutamate receptors, adenosine receptors, striatum

## Abstract

Dopamine D_2_ receptor antagonists modulate gene transcription in the striatum. However, the molecular mechanism underlying this effect remains elusive. Here we used the expression of *Nur77*, a transcription factor of the orphan nuclear receptor family, as readout to explore the role of dopamine, glutamate, and adenosine receptors in the effect of a dopamine D_2_ antagonist in the striatum. First, we investigated D_2_ antagonist-induced *Nur77* mRNA in D_2L_ receptor knockout mice. Surprisingly, deletion of the D_2L_ receptor isoform did not reduce eticlopride-induced upregulation of *Nur77* mRNA levels in the striatum. Next, we tested if an ibotenic acid-induced cortical lesion could block the effect of eticlopride on *Nur77* expression. Cortical lesions strongly reduced eticlopride-induced striatal upregulation of *Nur77* mRNA. Then, we investigated if glutamatergic neurotransmission could modulate eticlopride-induced *Nur77* expression. A combination of a metabotropic glutamate type 5 (mGlu5) and adenosine A_2A_ receptor antagonists abolished eticlopride-induced upregulation of *Nur77* mRNA levels in the striatum. Direct modulation of *Nur77* expression by striatal glutamate and adenosine receptors was confirmed using corticostriatal organotypic cultures. Taken together, these results indicate that blockade of postsynaptic D_2_ receptors is not sufficient to trigger striatal transcriptional activity and that interaction with corticostriatal presynaptic D_2_ receptors and subsequent activation of postsynaptic glutamate and adenosine receptors in the striatum is required. Thus, these results uncover an unappreciated role of presynaptic D_2_ heteroreceptors and support a prominent role of glutamate in the effect of D_2_ antagonists.

## Introduction

Dopamine antagonists have been used to alleviate schizophrenia symptoms for more than a half-century. There is a general agreement that *in vitro* binding affinity for dopamine D_2_ receptors predicts efficacy and likelihood of causing extrapyramidal side effects of antipsychotic drugs (Miyamoto et al., 2005). However, although all antipsychotic drugs used in clinic share the similar pharmacological profile of being D_2_ antagonists (with the exception of aripiprazole, which is a partial D_2_ agonist), the exact molecular and cellular mechanisms that convey their therapeutic and undesired effects remain elusive. A growing body of evidence indicate that drugs targeting other neurotransmitters, such as glutamate and adenosine, might also display antipsychotic activity (Lara et al., 2006; Conn et al., 2009; Krystal et al., 2010). But, because of the *in vivo* reciprocal functional relationships between dopamine, glutamate, and adenosine receptor activities, it is difficult to pin point the specific contribution of these different receptor subtypes.

The striatum expresses high levels of dopamine D_2_ receptors and is considered as an important brain area where dopamine and glutamate inputs are integrated to modulate psychomotor responses. The main striatal inputs are excitatory glutamatergic terminals coming from the cortex and thalamus, and dopamine afferences from the substantia nigra/ventral tegmental area complex. In order to process information coming from these multiple sources, striatal cells express a large array of neurotransmitter receptor subtypes at their surface (Gerfen and Surmeier, 2011). Striatal cells express glutamate receptors and a functional interaction between the activity of dopamine D_2_ and metabotropic glutamate receptors has been described. For example, blockade of metabotropic glutamate type 5 (mGlu5) receptor reduces haloperidol (D_2_ antagonist)-induced catalepsy (Ossowska et al., 2001). In addition, an interaction between dopamine D_2_, adenosine A_2A_, and mGlu5 receptors has been demonstrated in the striatum (Ferré et al., 2002; Kachroo et al., 2005; Cabello et al., 2009). For example, mGlu5 receptor antagonist-induced locomotor activity was abolished in postnatal forebrain-specific conditional (Cre/loxP system) A_2A_ receptor knockout mice (Kachroo et al., 2005). In addition, using combination of bimolecular fluorescence complementation and bioluminescence resonance energy transfer techniques, it has been shown that mGlu5, A_2A_, and D_2_ receptors can form oligomers in HEK293 cells (Cabello et al., 2009). High-resolution immunoelectron microscopy also indicated that the three receptors co-distribute within the extrasynaptic plasma membrane of the same dendritic spines of striatal synapses and co-immunoprecipitation experiments demonstrated the existence of an association of mGlu5, D_2_, and A_2A_ receptors in rat striatal homogenates (Cabello et al., 2009). Another important contributing factor to the striatal activity is the presence of neurotransmitter receptors located on cortical inputs to the striatum that regulate glutamate release. Indeed, activation of presynaptic D_2_ heteroreceptors located on glutamate neuron terminals (Schwarcz et al., 1978; Wang and Pickel, 2002) modulates glutamate release within the striatum and represents an important regulator of striatal excitatory inputs (Maura et al., 1988; Cepeda et al., 2001; Bamford et al., 2004).

Dopamine D_2_ receptors exist in two receptor isoforms derived from alternative splicing of the same gene (Giros et al., 1989). Accumulating evidence indicates that the D_2_ short (D_2S_) receptor isoform is mainly associated with presynaptic activities (Khan et al., 1998a; Usiello et al., 2000; Lindgren et al., 2003). Interestingly, it has been shown that dopaminergic modulation of corticostriatal glutamate release depended upon the D_2S_ receptors (Centonze et al., 2004b). On the other hand, the D_2_ long (D_2L_) receptor isoform seems to mediate postsynaptic dopamine receptor functions (Khan et al., 1998a; Usiello et al., 2000; Lindgren et al., 2003).

Modulation of gene transcription within neurons rapidly leads to protein synthesis and subsequent cellular adaptation. It is well documented that typical antipsychotic drugs rapidly induce genes like *c-fos*, Zif268, and *Nur77* (NGFI-B, Nr4a1; Herrera and Robertson, 1996; Herdegen and Leah, 1998; Lévesque and Rouillard, 2007). *Nur77* is a transcription factor of the nuclear receptor family that is rapidly induced after treatment with typical antipsychotic drugs (D_2_ antagonists; Beaudry et al., 2000; Maheux et al., 2005). Previous reports from our laboratory indicate that *Nur77* is involved in gene expression as well as in abnormal motor behaviors following exposure to a typical antipsychotic drug (for a review see, Lévesque and Rouillard, 2007). For example, haloperidol-induced striatal neuropeptides enkephalin and neurotensin mRNA expression is strongly impaired in *Nur77* knockout mice (Ethier et al., 2004a), while these mice display exacerbated haloperidol-induced vacuous chewing movements (similar to tardive dyskinesia; Ethier et al., 2004b). However, the exact contribution of glutamate, adenosine, and dopamine D_2S_ and D_2L_ receptors in the regulation of striatal gene expression remains to be clarified. In the present study, using *Nur77* mRNA expression as readout, we show that D_2_ antagonist-induced transcriptional activity in the striatum is mediated by interaction of the drug with presynaptic D_2S_ heteroreceptors located at corticostriatal terminals and subsequent activation of postsynaptic glutamate mGlu5 and adenosine A_2A_ receptors.

## Materials and Methods

### Animals

For pharmacological experiments, adult male wild type C57BL/6 mice (Charles River, St-Constant, QC, Canada) and mice lacking dopamine D_2L_ receptors [D_2L_ receptor knockouts, D_2L_(−/−)] and their littermates (Usiello et al., 2000) were used. All mice weighted 20–25 g and were housed five per cage in a temperature-controlled environment maintained under a 12-h light/dark cycle with *ad libitum* access to food and water. For experiments involving ibotenic acid lesions, we used male Sprague–Dawley rats (Charles River, St-Constant, QC, USA) weighing 280–320 g. Experimental procedures, including means to minimize discomfort, were reviewed and approved by the institutional Animal Ethics Committee of the Université de Montréal and were done in accordance with the Canadian Council on Animal Care guidelines for use of experimental animals.

### Drugs and treatments

The selective dopamine D_2_/D_3_ receptor antagonist eticlopride, cannabinoid CB_1_ receptor antagonist 1-(2,4-dichlorophenyl)-5-(4-iodophenyl)-4-methyl-*N*-1-piperidinyl-1H-pyrazole-3-carboxamide (AM251), muscarinic antagonist scopolamine, and selective adenosine A_2A_ antagonist SCH 58261 were purchased from Sigma–Aldrich (St. Louis, MO, USA). The selective mGlu5 antagonist 2-methyl-6-(phenylethynyl)pyridine hydrochloride (MPEP), mGlu1/5 agonist (RS)-3,5-Dihydroxyphenylglycine (DHPG), selective mGlu5 agonist (RS)-2-Chloro-5-hydroxyphenylglycine sodium salt (CHPG), selective A_2A_ agonist 4-[2-[[6-Amino-9-(*N*-ethyl-β-d-ribofuranuronamidosyl)-9H-purin-2-yl]amino]ethyl]benzene propanoic acid hydrochloride (CGS 21680), and excitatory amino acid transporters 1-2 (EAAT1-2) blocker (3S)-3-[[3-[[4-(Trifluoromethyl)benzoyl] amino]phenyl]methoxy]-l-aspartic acid (TBOA) were purchased from Tocris Bioscience (Avonmouth, UK). Non-competitive NMDA antagonists phencyclidine (PCP) and MK-801 were obtained from Sigma–Aldrich through a restricted importation permit. PCP analogs *N*-[1-(2-benzo(β)thiophenyl)cyclohexyl]piperidine (BTCP) and *N*-[1-(2-thiophenyl)cyclohexyl]piperidine (TCP) were obtained as a gift from Dr. J. M. Kamenka and the National Institute of Mental Health Chemical Synthesis Program (Rouillard et al., 1990).

First, we compared the effect of vehicle (NaCl 0.9%) and eticlopride (1 mg/kg) on striatal *Nur77* mRNA expression in wild type [D_2L_(+ / +)] and D_2L_(−/−) mice in order to assess the contribution of D_2S_ and D_2L_ receptor isoforms in the effect of the D_2_ antagonist. We used eticlopride, a highly selective D_2_/D_3_ receptor antagonist, because typical antipsychotic drugs used in clinics display a wider pharmacological profile, which could further complicate data interpretation. Note that eticlopride displays activities similar to typical antipsychotic drugs, but it is not used in clinic because of its poor pharmacokinetic properties (Martelle and Nader, 2008). Secondly, we assessed the effect of glutamatergic drugs on *Nur77* gene transcription. We acutely treated (0.25 ml, i.p.) five different groups of wild type mice (*N* = 5) as follows: (a) vehicle (NaCl 0.9%); (b) MK-801 (non-competitive NMDA receptor antagonist, 0.75 mg/kg); (c) PCP (5 mg/kg); (d) BTCP (16 mg/kg); and (e) TCP (0.75 mg/kg). Then, lower doses of MK-801 were used alone or in combination with eticlopride. Groups of mice were formed as follows: (a) vehicle (NaCl 0.9%; *N* = 6); (b) eticlopride (1 mg/kg; *N* = 5); (c) MK-801 (0.3 mg/kg; *N* = 5); (d) MK-801 (0.03 mg/kg; *N* = 5); (e) eticlopride (1 mg/kg) + MK-801 (0.3 mg/kg); (f) eticlopride (1 mg/kg) + MK-801 (0.03 mg/kg). MK-801 was injected 15 min before eticlopride. In the third experiment, involvement of mGlu5 and A_2A_ receptor drugs on eticlopride-induced *Nur77* upregulation was investigated. Animals were distributed into eight groups and treated as follows: (a) vehicles (NaCl 0.9 and 3% DMSO, 8% PEG600 in sterile water); (b) MPEP (mGlu5 antagonist, 10 mg/kg); (c) SCH58261 (A_2A_ antagonist, 5 mg/kg); (d) MPEP + SCH58261; (e) eticlopride (dopamine D_2_ antagonist, 1 mg/kg); (f) eticlopride (1 mg/kg) + MPEP (10 mg/kg); (g) eticlopride (1 mg/kg) + SCH58261 (5 mg/kg); (h) eticlopride (1 mg/kg) + MPEP + SCH58261 (*N* = 5 per group). MPEP and SCH58261 were administered 30 min before the dopamine antagonist. A similar paradigm was used to investigate the effect of the CB_1_ antagonist AM251 (5 mg/kg, i.p.) and muscarinic m1-4 antagonist scopolamine (2.5 mg/kg, i.p.) on eticlopride-induced *Nur77* expression. All the drug doses used were chosen based on previous studies using similar paradigms; eticlopride (Keefe and Adams, 1998; Pozzi et al., 2003; Bourhis et al., 2008), ionotropic glutamatergic drugs (Rouillard et al., 1990; Keefe and Adams, 1998; Chartoff et al., 1999), MPEP (Choe et al., 2002; Parelkar and Wang, 2004), SCH58261 (Pollack and Fink, 1995; Pinna et al., 1999), AM251 (Xi et al., 2006; Rubino et al., 2007), and scopolamine (Guo et al., 1992; Wang and McGinty, 1996). For all treatments, mice were sacrificed by decapitation 60 min after the last drug injection under CO_2_ anesthesia. Brains were rapidly removed, immediately immersed into cold 2-methylbutane (−40°C) for a few seconds and kept frozen at −80°C until used.

### *In situ* hybridization

Probe preparation and *in situ* hybridization of *Nur77* mRNA on brain slices were performed as previously reported (Beaudry et al., 2000; St-Hilaire et al., 2003; Maheux et al., 2005). Briefly, the *Nur77* single-stranded riboprobe was synthesized and labeled using Promega riboprobe kit (Promega, Madison, WI, USA), [^35^S]UTP (PerkinElmer Life and Analytical Sciences, Woodbridge, ON, Canada), and the RNA polymerase T_3_. *In situ* hybridization of the riboprobe with cryostat coronal brain sections (12 μm) mounted on Snowcoat X-tra slides (Surgipath, Winnipeg, MA, Canada) was done at 58°C overnight in a standard hybridization buffer containing 50% formamide. Brain sections were then apposed against BiomaxMR radioactive sensitive films (Eastman Kodak, New Haven, CT, USA) for 2 days.

### Quantification and statistical analysis

Levels of autoradiographic labeling on films were quantified by computerized densitometry as previously described (Beaudry et al., 2000; St-Hilaire et al., 2003; Maheux et al., 2005). Optical density of the autoradiograms was translated in nCi/g of tissue using [^14^C]-radioactivity standards (ARC 146A-^14^C standards, American Radiolabeled Chemicals Inc., St. Louis, MO, USA). *Nur77* mRNA levels were measured in the dorsomedial (StDM), dorsolateral (StDL), ventromedial (StVM), and ventrolateral (StVL) portions of the striatum, and the nucleus accumbens shell (AcSh) and core (AcC). The average level of labeling for each area was calculated from three to four adjacent brain sections of the same animal. Background intensity was subtracted from every measurement.

All data are expressed as group mean ± SEM and statistical analysis were performed using GraphPad Prism version 5.0 (GraphPad Software Inc., San Diego, CA, USA). Statistical comparisons between groups were obtained by using a one-way analysis of variance and a Bartlett’s test for equal variance. When the Bartlett’s test showed significant differences between variance, the log, or square root of data were used in the analysis. One-way analyses of variance were followed by a Tukey’s multiple comparison test as a *post hoc* test when appropriate.

### Dopamine D_2_ receptor autoradiography

Brain sections were pre-incubated in a 50 mM Tris-HCl buffer, pH 7.4, containing 120 mM NaCl, 5 mM KCl, 1 mM CaCl_2_, 1 mM MgCl_2_, 5.7 mM ascorbic acid, and 10 μM hydroxyquinoline for 15 min. The slides were then incubated for 1 h a room temperature in the same buffer containing 0.2 nM [^125^I]iodosulpride (Martres et al., 1985). Non-specific binding was evaluated in the presence of 2 μM raclopride. After 2 min rinses in ice-cold buffer, sections were dipped for 10 s in cold water, dried, and apposed to BioMax film. Autoradiograms were generated after an exposure time of 24 h.

### Ibotenic acid cortical lesions

Rats were anesthetized with isoflurane (2.5–3.5%, O_2_ 0.6 L/min) and mounted on a stereotaxic apparatus. The surface of cranium was exposed and the bone and *dura* above the left or right sensorimotor cortex was removed. A needle (300 μm in diameter) containing 5 μg/μl of ibotenic acid, or its vehicle, was inserted into the cortex at five anterior-posterior (0.5 mm apart) and 2–4 medial-lateral sites using the following flat skull coordinates: between 0.5 posterior and 1.5 mm anterior to Bregma; 1.4–3.3 mm lateral to the sagittal line, and 1.2–2.2 mm below the surface of the cortex. At each site, the needle was lowered to 2.2 mm below the cortex and a volume of 0.15 μl solution was injected over a minute; two (three for the most lateral and anterior sites) similar injections were made 0.5 and 1.0 mm above. The solution was injected using a microinfusion pump to activate a Hamilton microsyringe connected to the injection needle by polyethylene tubing. Similar saline injections were performed in sham animals. Ten days after surgery, animals were injected with eticlopride (1 mg/kg, i.p.) or saline and returned to their home cage. Sixty minutes after the injection, they were anesthetized with isoflurane (3.5%, O_2_ 0.6 L/min) and killed by decapitation. The brains were quickly removed dipped in cold 2-methylbutane and stored at −80°C.

### Corticostriatal organotypic slices

Corticostriatal organotypic slice cultures were prepared from 4–5 days old mice using the methods of Stoppini et al. (1991) and Stahl et al. (2009) with minor modifications (Stoppini et al., 1991; Stahl et al., 2009). Briefly, mouse brains were extracted and immerged in complete Hank’s buffer solution supplemented with glucose (5.6 mM) and sucrose (27.8 mM). Coronal 400 μm slices were cut using a McIlwain tissue chopper (Havard Apparatus, St. Laurent, QC, USA). Slices containing the striatum were transferred on Millicell filter inserts (0.4 μm; Millipore, Fisher scientific, Whitby, ON, USA) placed into a six-well plate filled with 1 ml of neurobasal medium containing 10% FBS, 1× N-2 supplement, 1× glutamine, 1× antibio-antimyc, and 0.6% glucose and maintained in culture for 3 days. Slices were then serum deprived for 14 h before pharmacological treatments. Drugs such as TBOA, DHPG, CHPG, and CGS 21680 were applied for 1 h directly to the culture medium. MPEP or quinpirole were applied 15 min prior subsequent treatments. Striata were removed using a glass Pasteur pipette as a tissue punches.

Striatal tissue samples were expelled directly in Trizol reagent for RNA extraction (Sigma–Aldrich, St. Louis, MO, USA). Reverse transcriptase reactions of 2 μg RNA were performed in a final volume of 20 μl using the High Capacity cDNA Reverse Transcription Kit with random primers (Applied Biosystems, Streetsville, ON, USA). Complementary DNA samples (1.5 μl) were used for SYBR green qPCR amplification of *Nur77* (Nr4a1; NM_010444.2) using Fast SYBR Green Master Mix (Applied Biosystems, Streetsville, ON, USA). Gene expression level for endogenous controls was determined using pre-validated Taqman Gene Expression Assays (Applied Biosystems, Streetsville, ON, USA). Four endogenous controls [glyceraldehyde-3-phosphate (GAPDH), hypoxanthine guanine phosphoribosyl transferase (HPRT1), β-actin, ACTB, and TATA binding protein (TBP)] were first assessed to determine which ones had the more stable expression in our experimental conditions. Further analysis of each sample was controlled using both GAPDH (NM_008084.2) and HPRT1 (NM_013556). The ABI PRISM^®^ 7900HT Sequence Detection System (Applied Biosystems, Streetsville, ON, USA) was used to detect amplification levels. All reactions were run in triplicate and average values of cycle thresholds (CTs) were used for quantification. The relative quantification of target genes was determined using the ∆∆CT method.

## Results

### Dopamine D_2L_ receptor knockout does not prevent eticlopride-induced *Nur77* expression in the striatum

**Figure 1:**
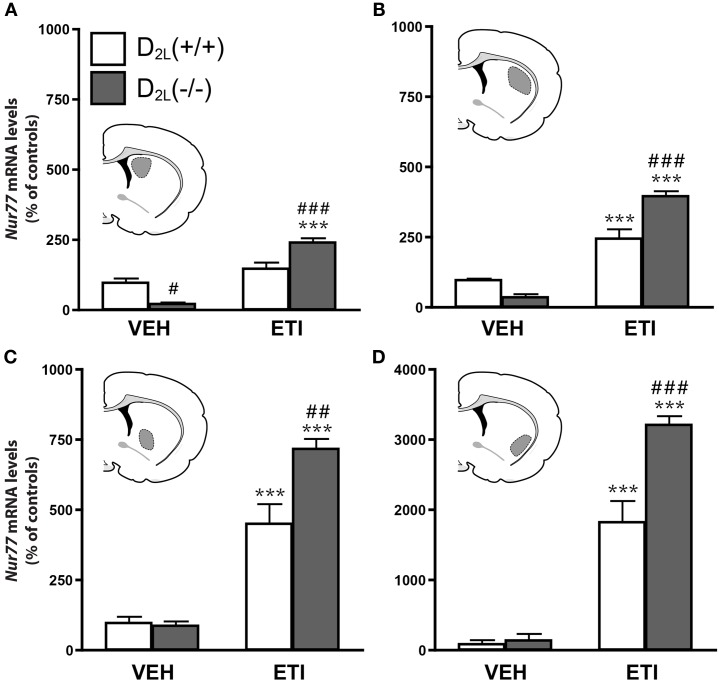
**Eticlopride induces *Nur77* mRNA levels in D_2L_ knockout mice**. *Nur77* mRNA levels (expressed as% of control) were measured in (**A**) dorsomedial (StDM), (**B**) dorsolateral (StDL), (**C**) ventromedial (StVM), (**D**) ventrolateral (StVL) portions of the striatum in dopamine D_2_ long receptor isoform knockout [D_2L_(−/−)] mice and their wild type littermates [D_2L_(+ / +)]. Histograms represent mean ± SEM (***p < 0.001 vs. vehicle (VEH) of the same strain, #*p* < 0.05 and ###*p* < 0.001 vs. eticlopride (ETI)-treated wild type [D_2L_(+ / +)] mice, *N* = 8 per group). Insets represent drawings of specific striatal areas (in gray) used for quantification.

We first investigated the contribution of D_2S_ and D_2L_ receptors in the modulation of *Nur77* mRNA expression in the striatum by comparing basal and eticlopride-induced *Nur77* mRNA levels in wild type (+/+) and D_2L_ knockout (−/−) mice (Usiello et al., 2000; Lindgren et al., 2003). D_2L_(−/−) mice displayed a significant reduction in basal *Nur77* mRNA levels in the dorsomedial striatum, but basal transcript levels were unchanged in all other striatal subterritories, as compared with their wild type littermates (Figure 1). As expected, administration of eticlopride in wild type mice led to a strong increase of *Nur77* transcript levels (Figure 1). Surprisingly, this modulation was still present in D_2L_ mutant mice (Figure 1), and was even significantly stronger when compared to their wild type littermates in most striatal subterritories (Figure 1). These data indicate that D_2L_ receptors might not play an important role in the effect of eticlopride-induced *Nur77* expression in the striatal tissue.

### Cortical lesions abolish eticlopride-induced *Nur77* expression in targeted striatal subterritorries

**Figure 2:**
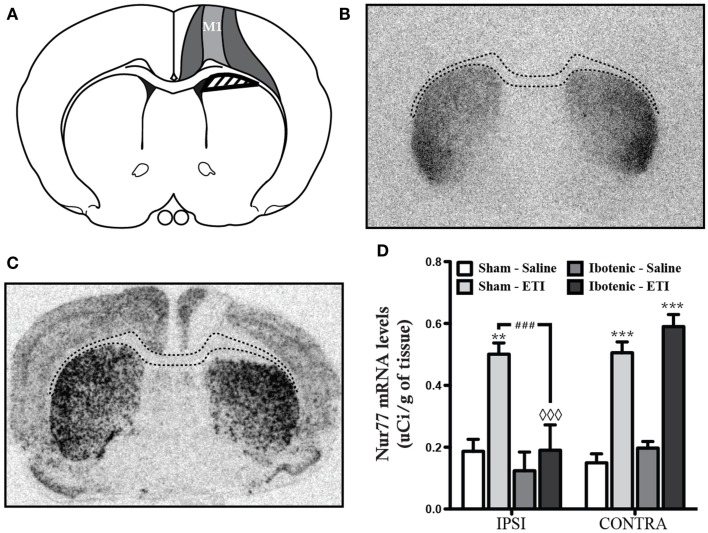
**Ibotenic acid lesions of the cortex prevent eticlopride-induced *Nur77* mRNA levels**. (**A**) Schematic representation showing the average extent of ibotenic acid lesions in the primary motor cortex (M1). The darker gray area represents the biggest lesions obtained, whereas lighter gray area shows the smallest ones. The shaded area indicates the region used for quantification of *Nur77* mRNA levels in the striatum. Similar quantifications were performed in the unlesioned side (contralateral). (**B**) Representative autoradioagram of [^125^I]iodosulpride specific binding at dopamine D_2_ receptors on a coronal slice showing that the lesions remain circumscribed in the cortex. Dash lines depict the position of the corpus callosum identified on an adjacent section after Nissl staining (not shown). (**C**) Representative autoradiogram showing *in situ* hybridization of the *Nur77* mRNA after eticlopride (ETI) administration. (**D**) Quantification of striatal *Nur77* mRNA levels in the ipsilateral (IPSI) and contralateral (CONTRA) sides of the lesion in animals treated with saline or eticlopride (ETI) and bearing a sham or an ibotenic acid-induced lesion. Data are expressed in μCi/g of tissue. Histogram bars represent means ± SEM (*N* = 5, **p < 0.01 and ***p < 0.001 vs. same side control (Sham-Saline); ###*p* < 0.001 vs. IPSI side of Ibotenic-ETI group and ◊◊◊*p* < 0.001 vs. CONTRA side of Ibotenic-ETI group).

In order to investigate whether integrity of the corticostriatal pathway, and therefore involvement of presynaptic D_2_ heteroreceptors in eticlopride-induced *Nur77* mRNA in the striatum, we proceeded to unilateral lesions of corticostriatal fibers using intra cortical injections of ibotenic acid. Ibotenic acid injections led to the lesion of most cortical neurons located in the primary motor cortex (M1) and affected all layers of the cortex. In some individuals, the lesion extended to the primary sensorimotor cortex (Figures 2A–C). Nissl staining (data not shown) and [^125^I] iodosulpride specific binding to dopamine D_2_ receptors showed that underlying subcortical regions were left intact (compared Figures 2B,C). As expected, eticlopride-induced a strong increase in *Nur77* mRNA levels in control animals bearing a sham lesion (Figure 2D). On the other hand, unilateral cortical lesion almost totally prevented eticlopride-induced upregulation of *Nur77* mRNA levels in the ipsilateral dorsal striatum (Figure 2D). Eticlopride-induced striatal *Nur77* mRNA levels were comparable to sham-lesioned animals in the contralateral side (Figure 2D). These data clearly indicate that the integrity of corticostriatal inputs is necessary for the upregulation of *Nur77* mRNA levels by the D_2_ antagonist in these striatal subterritories.

### Eticlopride-induced *Nur77* expression is reduced by glutamate mGlu5 and adenosine A_2A_ receptor antagonists

**Figure 3:**
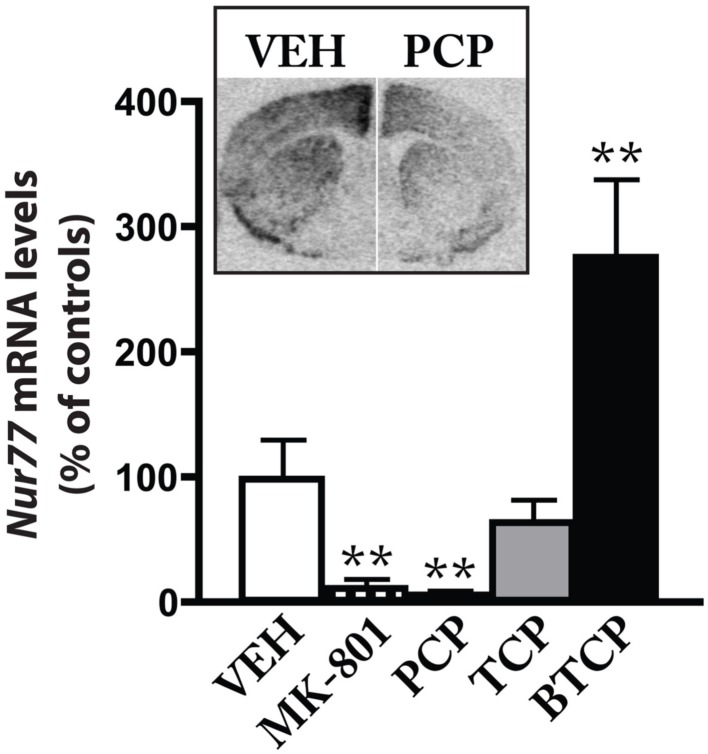
**NMDA antagonists reduced basal *Nur77* expression**. Levels of *Nur77* mRNA (expressed as% of control) were measured in the ventrolateral portion of the striatum after treatment with vehicle (VEH), MK-801 (0.75 mg/kg, i.p.), PCP (5 mg/kg, i.p.), TCP (0.75 mg/kg, i.p.), or BTCP (16 mg/kg, i.p.). Histograms represent mean ± SEM (**p < 0.01 vs. VEH, *N* = 5 per group). Inset: representative autoradiograms showing *Nur77* mRNA levels in a left hemi-brain from the VEH group and a right hemi-brain from the PCP group.

We then investigated if glutamate receptors located on postsynaptic striatal medium spiny neurons might contribute to the effect of the D_2_ antagonist. To test this hypothesis, we combined the administration of eticlopride with ionotropic or metabotropic glutamate receptor antagonists. Although the modulation of *Nur77* mRNA levels by dopamine D_2_ antagonists has been previously described (Beaudry et al., 2000; Werme et al., 2000; Maheux et al., 2005), modulation of this transcription factor by glutamate receptors remains largely unexplored. Thus, we first evaluated the effect of NMDA antagonists on striatal *Nur77* mRNA levels in wild type mice. Both MK-801 (0.75 mg/kg) and PCP (5 mg/kg) strongly reduced basal *Nur77* mRNA levels in the striatal complex (Figure 3). We also used two PCP analogs to confirm the role of NMDA receptors in the effect of PCP. TCP is a PCP analog, which display a stronger affinity for the PCP site on the NMDA receptor than PCP itself, whereas BTCP is a PCP analog characterized by a higher affinity for the dopamine transporter (Rouillard et al., 1990). A dose equivalent of BTCP produced a strong increase in *Nur77* expression (Figure 3). This effect is consistent with the BTCP psychostimulant-like property, since it has been previously shown that psychostimulants increased *Nur77* mRNA levels (Bäckman and Morales, 2002; Bhardwaj et al., 2003). A PCP dose equivalent of TCP based on their affinity for the PCP site did not induce *Nur77* mRNA levels, but rather tended to reduce it (however, it did not reach significance). Thus, we may conclude from these experiments that basal striatal *Nur77* expression is under a tonic control by NMDA receptors.

**Figure 4:**
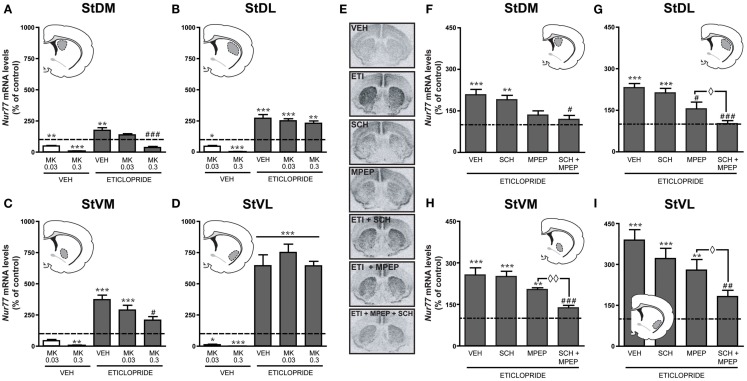
**Blockade of mGlu5 and A_2A_ receptors abolishes eticlopride-induced upregulation of *Nur77* mRNA expression**. Modulation of basal and eticlopride-induced *Nur77* mRNA levels by the NMDA antagonist MK-801 (0.03 or 0.3 mg/kg) was measured in (**A**) dorsomedial (StDM), (**B**) dorsolateral (StDL), (**C**) ventromedial (StVM), (**D**) ventrolateral (StVL) portions of the striatum. Insets represent drawings of specific striatal areas (in gray) used for quantifications. Basal *Nur77* mRNA levels in respective striatal subterritories of untreated animals were set to 100% and are indicated as dash lines. Histograms represent mean ± SEM of animals treated with MK-801 (0.3 or 0.03 mg/kg) alone or eticlopride- and MK-801 (MK)-treated animals (*p < 0.05, **p < 0.01, and ***p < 0.001 vs. vehicle (VEH); #*p* < 0.05 and ###*p* < 0.001 vs. eticlopride, *N* = 5 per group). (**E**) Representative autoradiograms showing *Nur77* mRNA levels following drug administrations. Inhibitions of eticlopride-induced *Nur77* expression (expressed as% of control, dash lines) by the vehicle (VEH), SCH58261 (SCH), MPEP, or SCH + MPEP were measured in (**F**) dorsomedial (StDM), (**G**) dorsolateral (StDL), (**H**) ventromedial (StVM), (**I**) ventrolateral (StVL) portions of the striatum. *Nur77* mRNA levels in animal treated with VEH, MPEP, SCH58261, or MPEP + SCH58261 alone can be found in Table 1. Histograms represent mean ± SEM of eticlopride-treated groups (**p < 0.01 and ***p < 0.001 vs. control; #*p* < 0.05, ##*p* < 0.01, and ###*p* < 0.001 vs. eticlopride alone (VEH); ⋄*p* < 0.05 and ⋄⋄*p* < 0.01 vs. MPEP + eticlopride, *N* = 5 per group).

In the next step, we investigated the role of NMDA receptors into the upregulation of *Nur77* mRNA expression induced by the D_2_ antagonist eticlopride. Since the initial dose of MK-801 (0.75 mg/kg) induced a strong reduction of basal *Nur77* expression (Figure 3), which could be mixed up with the putative effect of NMDA on eticlopride-induced *Nur77* expression, we investigated two lower doses of MK-801 (0.03 and 0.3 mg/kg) alone or in combination with eticlopride (Figures 4A–D). At these concentrations, MK-801 was still able to reduce basal *Nur77* levels, but to a lesser extent (Figures 4A–D). However, both MK-801 doses did not alter eticlopride-induced *Nur77* expression in lateral striatal subterritories (Figures 4B,D) and the lower dose of MK-801 (0.03 mg/kg) also remained without effect in medial portions of the striatum (Figures 4A,C). Note that a similar low dose of MK-801 can modulate *c-fos* mRNA levels in the brainstem, as well as to reduce reward threshold induced by electrical self-stimulation of the ventral tegmental area (Hattori et al., 2004; Clements and Greenshaw, 2005), indicating that this dose is effective.

Since these results suggested a modest contribution of NMDA receptors in eticlopride-induced *Nur77* mRNA levels in the striatum, we investigated the role of the metabotropic glutamate mGlu5 receptor and its partner, the adenosine A_2A_ receptor (Figures 4E–I). Administration of MPEP (mGlu5 antagonist) or SCH58261 (A_2A_ antagonist) alone had no effect on *Nur77* mRNA expression in the brain areas analyzed (Table 1). When co-administered with eticlopride, SCH58261 had no significant effect on the upregulation of *Nur77* mRNA expression (Figures 4E–I). Selective blockade of mGlu5 receptor with MPEP tended to reduce eticlopride-induced *Nur77* mRNA levels, but this effect reached significance only in the dorsolateral portion of the striatum (Figure 4G). Interestingly, co-administration of both MPEP and SCH58261 strongly reduced eticlopride-induced *Nur77* mRNA levels in all striatal subterritories, restoring *Nur77* mRNA levels back to baseline (Figures 4E–I).

**Table 1:** ***Nur77* mRNA levels following vehicle, MPEP, and SCH58261 in striatal subterritories**.

Brain areas	*Nur77* mRNA levels (% of control)			
	VEH	MPEP	SCH58261	MPEP + SCH58261
StDM	100 ± 15	86 ± 20	83 ± 7	83 ± 4
StDL	100 ± 12	105 ± 22	93 ± 18	87 ± 4
StVM	100 ± 14	155 ± 21	77 ± 29	129 ± 22
StVL	100 ± 16	119 ± 26	75 ± 50	108 ± 35
AcSh	100 ± 10	88 ± 36	73 ± 5	107 ± 16
AcC	100 ± 11	141 ± 13	98 ± 14	106 ± 9

*AcC, nucleus accumbens core; AcSh, nucleus accumbens shell; StDM, dorsomedial striatum; StDL, dorsolateral striatum; StVM, ventromedial striatum; StVL, ventrolateral striatum; VEH, vehicle*.

**Figure 5:**
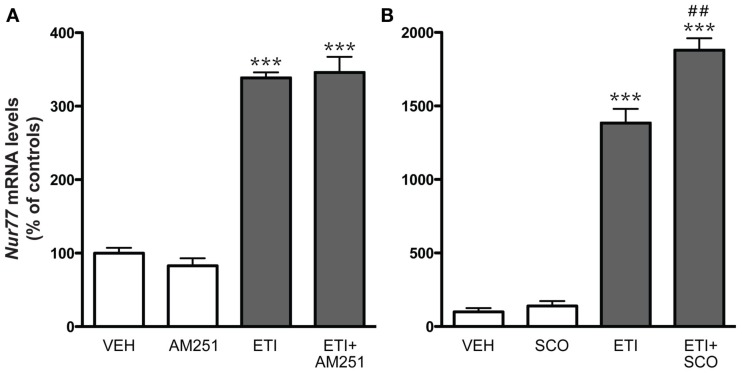
**Cannabinoid and muscarinic receptor antagonists did not reduce eticlopride-induced *Nur77* mRNA levels in the striatum**. (**A**) Groups of mice were treated with, either saline 0.9% (VEH, *N* = 5), eticlopride (ETI, 1 mg/kg, *N* = 5), CB_1_ antagonist AM251 (5 mg/kg, *N* = 5), or a combination of ETI and AM251. (**B**) Groups of mice were treated with, either VEH (*N* = 5), ETI (1 mg/kg, *N* = 5), scopolamine (SCO, 2.5 mg/kg, *N* = 5), or a combination of SCO and ETI. AM251 and SCO were administered 15 min prior ETI. Histograms represent means ± SEM. *Nur77* mRNA levels are expressed in% of control (VEH) in the ventrolateral portion of the striatum (***p < 0.001 vs. VEH and ##*p* < 0.01 vs. ETI alone). Similar results were obtained in the other striatal subterritories (data not shown).

To complement the pharmacological characterization of the dopamine D_2_ receptor antagonist effect, we also investigated the contribution of cannabinoid and muscarinic drugs in the modulation of striatal *Nur77* mRNA levels induced by eticlopride. Systemic injections of AM251, a CB_1_ receptor antagonist or scopolamine, a muscarinic m1-4 receptor antagonist, alone did not modulate *Nur77* mRNA levels in the ventrolateral portion of the striatum (Figure 5). Co-administration of AM251 or scopolamine with the D_2_ antagonist did not reduce eticlopride-induced *Nur77* expression in the ventrolateral portion of the striatum (Figure 5). In fact, scopolamine significantly further increased eticlopride-induced *Nur77* expression (Figure 5).

### mGlu5 receptors, but not D_2_ receptors, modulate *Nur77* expression in striatal organotypic cultures

**Figure 6:**
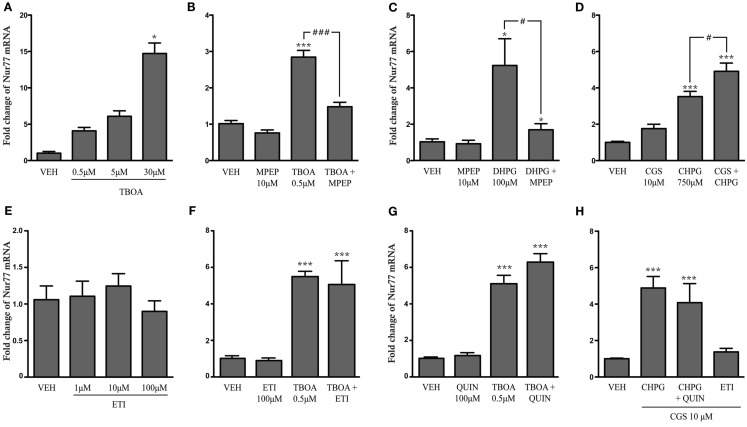
**Striatal *Nur77* expression is induced by metabotropic glutamate and adenosine receptors in corticostriatal organotypic slice cultures**. *Nur77* mRNA levels were measured in striatal tissue punches using quantitative real-time RT-PCR from corticostriatal organotypic cultures treated with (**A**) vehicle (VEH) or increasing doses of TBOA, a glutamate reuptake inhibitor (*N* = 3; *p < 0.05 vs. VEH group), (**B**) VEH, TBOA, MPEP (a mGlu5 receptor antagonist) alone, or TBOA + MPEP (*N* = 5–6; ***p < 0.001 vs. VEH; ###*p* < 0.001 vs. TBOA group), (**C**) VEH, MPEP, DHPG (a mGlu1/5 receptor agonist), or MPEP + DHPG (*N* = 5; *p < 0.05 vs. VEH; #*p* < 0.05 vs. DHPG group), (**D**) VEH, CGS21680 (CGS, a adenosine A_2A_ receptor agonist), CHPG (a selective mGlu5 receptor agonist), and CGS + CHPG (*N* = 5; ***p < 0.001 vs. VEH; # *p* < 0.05 vs. CHPG group), (**E**) VEH or increasing doses of the D_2_ receptor antagonist eticlopride (ETI; *N* = 6), (**F**) VEH, quinpirole (QUIN, a dopamine D_2_ receptor agonist), ETI, TBOA, TBOA + QUIN (*N* = 6–9; ***p < 0.001 vs. VEH group), (**G**) VEH, ETI, TBOA, and TBOA + ETI (*N* = 3; ***p < 0.001 vs. VEH group), and (**H**) VEH, CHPG + CGS, CHPG + CGS + QUIN, and CGS + ETI (*N* = 3; ***p < 0.001 vs. VEH). Data are represented as fold change of *Nur77* mRNA levels compared to controls (VEH) and normalized with GAPDH house keeping transcript levels. Similar results were obtained using normalization with the HPRT1 housekeeping gene (data not shown).

To directly demonstrate the contribution of mGlu5 and adenosine A_2A_ receptor subtypes to the modulation of *Nur77* mRNA expression, we tested whether an increase of glutamatergic neurotransmission or mGlu5 and A_2A_ receptor activation can modulate *Nur77* mRNA levels in corticostriatal organotypic slices in culture. To this end, we used TBOA, which is a high affinity blocker of glial excitatory amino acid transporters 1 and 2 (EAAT1-2), which has been shown to increase glutamate concentration in acute slice preparations (Beurrier et al., 2009). TBOA alone produced a nice and strong dose-dependent *Nur77* mRNA induction (Figure 6A). Pre-treatment of organotypic cultures with MPEP, a selective mGlu5 receptor antagonist, significantly reduced the effect of the low dose of TBOA on *Nur77* mRNA induction, confirming the important role of mGlu5 receptor in the induction of *Nur77* gene transcription by glutamate (Figure 6B). We therefore tested direct activation of mGlu5 receptor in organotypic corticostriatal slices. Exposition to DHPG, a mGlu1/5 agonist, significantly increased *Nur77* mRNA expression in the striatum by approximately fivefold (Figure 6C). This effect was selective for mGlu5 receptors since co-administration of MPEP led to a complete blockade of DHPG-induced *Nur77* mRNA upregulation (Figure 6C). Additionally, activation of mGlu5 receptor with a more specific agonist (CHPG) also led to a strong increase of *Nur77* mRNA levels (Figure 6D). As previously observed *in vivo*, this effect can be potentiated by a concomitant activation of A_2A_ receptors with CGS21680 (Figure 6D). Direct exposure of corticostriatal organotypic slices to eticlopride or quinpirole (dopamine D_2_ agonist) alone or in combination with TBOA did not modulate *Nur77* mRNA levels (Figures 6E–G). In addition, both D_2_ agonist and antagonist drugs were not able to modulate mGlu5/A_2A_ agonist-induced striatal *Nur77* mRNA levels in organotypic slice preparations (Figure 6H). These results suggest that postsynaptic D_2_ receptors do not significantly contribute to striatal transcriptional activity in the present experimental conditions.

## Discussion

**Figure 7:**
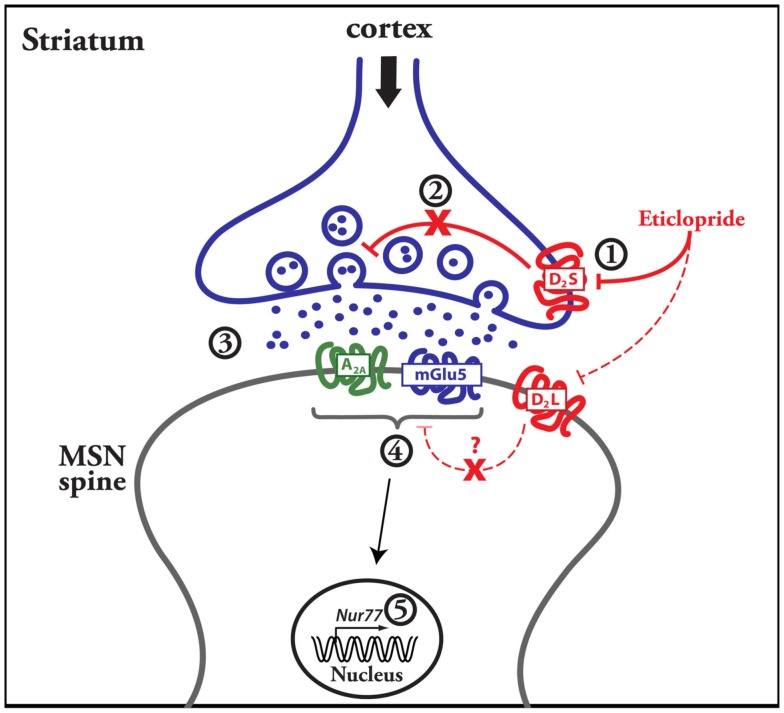
**Suggested events leading to the modulation of striatal gene transcription by a dopamine D_2_ antagonist**. The figure includes a schematic representation of a striatal medium spiny neuron (MSN) with a glutamatergic terminal coming from the cortex. Data presented herein suggest that the dopamine D_2_ antagonist blocks presynaptic D_2_ heteroreceptors (D_2S_) located on glutamatergic terminals, which control glutamate release (step 1 and 2). This leads to an elevation of glutamate contents in the synapse (step 3), which in turn activate postsynaptic metabotropic mGlu5 and adenosine A_2A_ receptors (step 4). Intracellular signaling events associated with activation of mGlu5 receptors (in addition to adenosine A_2A_ receptors) then lead to the increase of *Nur77* gene transcription in MSNs (step 5). The dash line and question mark that link postsynaptic D_2L_ and A_2A_-mGlu5 receptors illustrate the fact that we were unable to show a contribution of postsynaptic dopamine D_2_ receptors in the transcriptional effect of mGlu5/A_2A_ receptor agonists in our organotypic slice preparations.

Our results indicate that the modulation of striatal *Nur77* mRNA expression by a dopamine D_2_ antagonist depends on the integrity of the corticostriatal pathway and postsynaptic striatal mGlu5 and A_2A_ receptors activation (see Figure 7 for a proposed model). Thus, presynaptic modulation of glutamate neurotransmission is required in the modulation of striatal transcriptional activity following the administration of a selective D_2_ receptor antagonist (see Figure 7).

It is generally recognized that blockade of striatal postsynaptic D_2_ receptors is associated with antipsychotic drug activity in the striatum. Thus, the preservation of the effect of the D_2_ antagonist on *Nur77* mRNA expression in the D_2L_(−/−) mice was somewhat surprising. In the D_2L_(−/−) mouse, the lack of exon 6, specific for the D_2L_ isoform, results in the conversion of all dopamine D_2_ receptor transcripts into D_2S_ receptors (Usiello et al., 2000; Lindgren et al., 2003; Centonze et al., 2004b). Our results clearly show that the D_2_ receptor antagonist could still induce strong activation of *Nur77* gene transcription in D_2L_ mutant mice. Therefore, ectopic postsynaptic D_2S_ expression might have fulfilled D_2L_ activity in D_2L_(−/−) mice. However, this possibility is unlikely because postsynaptic activity of dopamine, such as modulation of DARPP-32 phosphorylation in the striatum, is hampered in this mouse strain (Lindgren et al., 2003). A better understanding of the difference between the two isoforms subcellular expression will be required to better explain this observation. Since D_2L_ receptors are mainly associated with postsynaptic effect of dopamine (Khan et al., 1998b; Usiello et al., 2000; Lindgren et al., 2003), it suggests that a presynaptic event might be needed for eticlopride-induced gene transcription in the striatum.

Dopamine D_2_ receptors can modulate glutamate signaling through both pre- and postsynaptic mechanisms. These receptors can be found on cortical inputs from the corticostriatal pathway (presynaptic D_2_ heteroreceptors), where they can modulate glutamate release in the striatum (Bamford et al., 2004; Higley and Sabatini, 2010). The present results show that the integrity of the corticostriatal pathway and therefore the presence of presynaptic D_2_ heteroreceptors are essential for eticlopride-induced striatal gene expression. We demonstrated this by performing extensive lesions of motor cortex by means of intra cortical administration of ibotenic acid. Such lesions have been documented to decrease the role of excitatory amino acid transmission in targeted subcortical areas (Cromwell and Levine, 1996; Garcia et al., 2010). In accordance, the striatal areas altered by the present cortex lesions were restricted to striatal subterritorries (Voorn et al., 2004; Garcia et al., 2010). This suggests that the corticostriatal pathway is of the upmost importance in mediating D_2_ antagonist regulation of transcription in those striatal areas.

These results fall in line with increasing evidence showing the importance of glutamate-dopamine interplay in striatal functions. Interestingly, the present results indicate that ionotropic NMDA receptors are minimally involved in the upregulation of striatal gene transcription by the D_2_ antagonist, but rather support an important role for the metabotropic mGlu5 receptor subtype. Noteworthy, a number of reports have shown an important interaction between dopamine D_2_ and mGlu5 receptors along with adenosine A_2A_ receptors. First, an intracellular signaling synergy has been observed between mGlu5 and adenosine A_2A_ receptors in the striatum (Nishi et al., 2003). It has been shown also that co-activation of these receptors can induce *c-fos* expression in a synergistic manner in the nucleus accumbens and dual blockade of these receptors leads to a synergistic activation of locomotor activity (Ferré et al., 2002; Kachroo et al., 2005). Our data (both *in vivo* and *in vitro*) reveal another system in which activation of mGlu5 and A_2A_ produces an additive response. Although previous observations support a direct postsynaptic interaction between D_2_, A_2A_, and mGlu5 receptors (Ferré et al., 2002; Kachroo et al., 2005; Bertran-Gonzalez et al., 2009), the present observations suggest that D_2_-A_2A_-mGlu5 interaction may also occur indirectly from the activity of a presynaptic D_2_ receptor (see Figure 7). Presynaptic modulation of glutamate neurotransmission by a D_2_ antagonist is also consistent with previous studies showing that acute and chronic administration of typical antipsychotic drugs, including haloperidol and eticlopride, increase glutamate concentration in the striatum (Bardgett et al., 1993; Yamamoto and Cooperman, 1994).

Experiments using organotypic corticostriatal slices confirm the direct modulation of *Nur77* expression by mGlu5 and A_2A_ receptors, which is consistent with results obtained in the hippocampus (Lindecke et al., 2006). In acute slice experiments, the glutamate uptake inhibitor TBOA can induce changes in postsynaptic currents (Beurrier et al., 2009) and quinpirole could decrease excitatory postsynaptic potential triggered by TBOA and low frequency cortical stimulation (Yin and Lovinger, 2006). However, we were not able to modulate *Nur77* mRNA levels with quinpirole in our organotypic cultures (with or without TBOA). This suggests that corticostriatal terminals might not spontaneously release enough glutamate in our organotypic slice preparations to record a presynaptic effect of the dopamine D_2_ receptor agonist. In addition, direct D_2_ receptor activation with quinpirole, or blockade by eticlopride, in organotypic cultures also remained ineffective. This suggests that postsynaptic D_2_ receptor activity is not associated with the modulation of *Nur77* expression in striatal cells. Thus, these results support an indirect effect of D_2_ receptor drugs on striatal gene expression (Figure 7).

The contribution of other presynaptic D_2_ heteroreceptors, such as those located on striatal cholinergic interneurons (Yan et al., 1997; Tozzi et al., 2011) is unlikely because we showed that the muscarinic antagonist scopolamine administration did not reduce eticlopride-induced *Nur77* mRNA expression in the striatum. On the contrary, scopolamine potentiated the effect of eticlopride. This might reflect a prominent activity of this non-selective muscarinic antagonist at m1–2 receptor subtypes located on corticostriatal terminals, which can also modulate glutamate release (Alcantara et al., 2001; Higley et al., 2009). The participation of presynaptic D_2_ autoreceptors located on dopamine terminals is unlikely as well. Indeed, an elegant study recently showed that mice specifically lacking D_2_ autoreceptors (autoDrd2KO) display similar haloperidol-induced reduction of horizontal locomotor activity compared to their littermate (Bello et al., 2011), indicating that these receptors are not involved in this D_2_ antagonist-induced effect. But, transcriptional activity of haloperidol has not been investigated in this transgenic model. Contribution of the dopamine D_3_ receptor, which is also a potent target of eticlopride, can be discarded as well because haloperidol-induced gene expression in the striatum is not altered in D_3_ receptor knockout mice (Robertson et al., 2004) and D_3_ receptor specific antisense oligonucleotides, which significantly reduced D_3_ receptor binding site density, has been shown to reduce *Nur77* expression (Tremblay et al., 1999). CB_1_ receptors can regulate glutamate release from corticostriatal terminals (Gerdeman and Lovinger, 2001; Kofalvi et al., 2005) and a D2-like-CB_1_ receptor interplay has been observed in the striatum (Centonze et al., 2004a). Thus, modulation of the present glutamate-dependent effect of eticlopride by CB_1_ remains a possibility. Although we showed that cannabinoid CB_1_ receptors antagonist AM251 did not modulate eticlopride-induced striatal *Nur77* mRNA expression and that similar AM251 dosages can modulate dopamine-related signaling (Kofalvi et al., 2005; Corbillé et al., 2007), we cannot discard the possibility that a higher dose of AM251 might have produced an effect. In addition, a contribution of other receptor subtypes such as glutamate mGlu2/3 receptors (Garcia et al., 2010), which can also modulate glutamate neurotransmission at corticostriatal terminals, or from thalamostriatal afferences in the modulation of *Nur77* expression in the striatal complex cannot be excluded. Additional experiments will be required in order to investigate the exact contribution of these receptor subtypes and striatal afferents to D_2_ receptor antagonist-induced gene transcription in selective striatal subterritories.

In conclusion, the present data indicates that mGlu5-A_2A_ activities mediate D_2_ receptor antagonist effect on striatal gene expression (Figure 7). Our results provide new evidences that adenosine and glutamate receptors might play an important role in typical antipsychotic drug-mediated effects and uncover an unappreciated role of presynaptic D_2_ heteroreceptors into the effect of dopamine D_2_ receptor antagonists. In addition, these results suggest that some striatal intracellular signaling events following D_2_ antagonist treatments might be attributed to metabotropic glutamate and adenosine receptor activities instead of dopamine D_2_ receptors.

## Conflict of Interest Statement

The authors declare that the research was conducted in the absence of any commercial or financial relationships that could be construed as a potential conflict of interest.
